# The Omega-3 Docosahexaenoyl Ethanolamide Reduces CCL5 Secretion in Triple Negative Breast Cancer Cells Affecting Tumor Progression and Macrophage Recruitment

**DOI:** 10.3390/cancers15030819

**Published:** 2023-01-29

**Authors:** Giuseppina Augimeri, Marco Fiorillo, Catia Morelli, Salvatore Panza, Cinzia Giordano, Ines Barone, Stefania Catalano, Diego Sisci, Sebastiano Andò, Daniela Bonofiglio

**Affiliations:** 1 Department of Pharmacy, Health and Nutritional Sciences, University of Calabria, Via P. Bucci, Arcavacata di Rende (CS), 87036 Cosenza, Italy; 2 Centro Sanitario, University of Calabria, Via P. Bucci, Arcavacata di Rende (CS), 87036 Cosenza, Italy

**Keywords:** omega-3 polyunsaturated fatty acids, omega-3 polyunsaturated fatty acid amides, breast cancer, triple-negative breast cancer cells, peroxisome proliferator-activated receptor gamma, C-C motif chemokine ligand 5, tumor-associated macrophages

## Abstract

**Simple Summary:**

Triple-negative breast cancer (TNBC) is associated with a generally poor breast cancer outcome, intensifying the need for novel therapeutic strategies beyond chemotherapy for this challenging subtype of breast cancer. Evidence shows that omega-3 polyunsaturated fatty acids and their derivatives exert antineoplastic effects within the breast tumor microenvironment, highlighting their potential as anticancer agents. Here, we demonstrated that docosahexaenoic acid conjugated with ethanolamine (DHEA) is able to attenuate the malignant phenotype of TNBC cells, reducing cell migration and invasion and affecting their metabolic profile through a shift from an energetic to a quiescent state. In a co-culture system, TNBC cells exposed to DHEA suppressed macrophage recruitment and cell viability along with the expression of genes related to tumor-associated macrophage (TAM) phenotype. Intriguingly, we unraveled that DHEA effects were mediated by CCL5 secretion from TNBC cells, highlighting this molecule as a promising treatment option against TNBC.

**Abstract:**

Triple-negative breast cancer (TNBC), an aggressive breast cancer subtype lacking effective targeted therapies, is considered to feature a unique cellular microenvironment with high infiltration of tumor-associated macrophages (TAM), which contribute to worsening breast cancer patient outcomes. Previous studies have shown the antitumoral actions of the dietary omega-3 docosahexaenoic acid (DHA) in both tumor epithelial and stromal components of the breast cancer microenvironment. Particularly in breast cancer cells, DHA can be converted into its conjugate with ethanolamine, DHEA, leading to a more effective anti-oncogenic activity of the parent compound in estrogen receptor-positive breast cancer cells. Here, we investigated the ability of DHEA to attenuate the malignant phenotype of MDA-MB-231 and MDA-MB-436 TNBC cell lines, which in turn influenced TAM behaviors. Our findings revealed that DHEA reduced the viability of TNBC cells in a concentration-dependent manner and compromised cell migration and invasion. Interestingly, DHEA inhibited oxygen consumption and extracellular acidification rates, reducing respiration and the glycolytic reserve in both cell lines. In a co-culture system, TNBC cells exposed to DHEA suppressed recruitment of human THP-1 cells, reduced their viability, and the expression of genes associated with TAM phenotype. Interestingly, we unraveled that the effects of DHEA in TNCB cells were mediated by reduced C-C motif chemokine ligand 5 (CCL5) expression and secretion affecting macrophage recruitment. Overall, our data, shedding new light on the antitumoral effects of DHA ethanolamine-conjugated, address this compound as a promising option in the treatment of TNBC patients.

## 1. Introduction

Triple-negative breast cancer (TNBC) represents the most aggressive type of breast cancer, characterized by a high rate of recurrence and the worst outcomes. Since TNBC does not express the estrogen receptor, the progesterone receptor, and the human epidermal growth factor receptor 2, limited, targeted specific agents are available for the treatment of TNBC, which is usually managed by conventional approaches, such as surgery, chemo and radiotherapy, resulting in a high rate of breast cancer recurrence [1]. Specifically, PARP inhibitors can be used in patients harboring *BRCA* mutation, while immunotherapy alone or in combination with chemotherapy can represent additional pharmacological tools for Programmed Cell Death Ligand 1 (PD-L1) positive metastatic TNBC patients [2]. Thus, research is ongoing to identify new targets and treatment options for this type of breast cancer.

The tumor microenvironment (TME) plays an important role in the development of breast cancer, influencing the efficacy of therapeutic strategies and patient outcomes [3,4]. TME is composed of several cell types, including immune cells, blood vessels, and structural cells like fibroblasts, as well as extracellular matrix molecules, growth factors, and other signaling molecules [5].

Tumor-associated macrophages (TAMs) are one of the main components of the breast TME, representing up to 50% of the tumor mass [6]. They derive from circulating monocytes, and their infiltration in the TME is associated with a poor prognosis. Indeed, TAMs promote tumor growth, progression, and metastasis and are considered important players in the modulation of angiogenesis, immune suppression, and inflammation [7]. Moreover, depending on their microenvironmental cues, TAMs can acquire different phenotypes characterized by increased production of pro-inflammatory cytokines that may exert anti-tumor properties, sustaining the recruitment of other immune cells to the tumor and suppressing the anti-tumor immune response [8]. Particularly, breast cancer cells secrete factors that support the recruitment of monocyte in the TME and their differentiation and polarization in TAMs [9]. Understanding the complex interplay between breast cancer cells and TAMs that regulate their interaction represents an important area of research, as it may provide novel insight into therapeutic strategies against breast cancer [10,11,12].

The omega-3 polyunsaturated fatty acids (PUFAs), docosahexaenoic acid (DHA) and eicosapentaenoic acid (EPA), have been demonstrated to exert potential beneficial action in breast cancer prevention and treatment [13,14,15]. Dietary consumption of DHA and EPA, mainly found in fatty fish, such as salmon, mackerel, herring, and sardines, but also in walnuts, pumpkin seeds, flaxseeds, and soya beans reduces the risk of breast cancer and counteracts the side effects of chemotherapy in survivors [16,17]. Interestingly, it has been reported that DHA is a more potent inhibitor of breast cancer metastasis than EPA [18]. Moreover, it has been found that DHA can be converted by breast cancer cells in a conjugate with ethanolamine, DHEA, which results in more active metabolite than parental compounds [19,20]. Our previous studies showed that DHEA induces anti-proliferative actions through the activation of the autophagy in estrogen receptor alpha-positive breast cancer cells in a peroxisome proliferator-activated receptor gamma (PPARγ) dependent manner [21]. Here, we aim to explore the effects of DHEA in TNBC cells and its impact in modulating macrophage recruitment and TAM phenotype in the breast tumor microenvironment.

## 2. Materials and Methods

### 2.1. Cell Cultures

Human MDA-MB-231 and MDA-MB-436 TNBC cells and the human THP-1 monocytic cell line (American Type Culture Collection, Manassas, VA, USA) were authenticated and stored according to the supplier’s instructions. Cells were used within 4 months after recovery of frozen aliquots and regularly tested for mycoplasma-negativity (MycoAlert Mycoplasma Detection Assay, Lonza, Basilea, Basel, Switzerland). Human monocytic THP-1 cells were incubated with 100 nM phorbol 12-myristate 13-acetate (PMA) for 12 h to induce the differentiation into mature macrophage-like cells. Then, differentiated macrophages were maintained in full media for 24 h prior to incubation for 5 days with the conditioned media (CM) from TNBC cells.

### 2.2. Cell Viability Assay

Cell viability was assessed by (3-(4,5-Dimethylthiazol-2-yl)-2,5-Diphenyl-tetrazolium-Bromide) (MTT, Sigma-Aldrich, Milan, Italy), as described [21] and expressed as percentage respect to vehicle-treated cells. IC_50_ values were calculated using GraphPad Prism 7 (GraphPad Prism software, Inc., San Diego, CA, USA).

### 2.3. Wound-Healing Assays

TNBC cells were treated with vehicle or DHEA 10 µM, and cell migration was monitored within 24 h. The rate of wound healing was quantified as previously reported [22]. Images were acquired by the OLYMPUS-BX51 microscope (Olympus, Tokyo, Japan).

### 2.4. Transmigration Assays

TNBC cells, treated with vehicle or DHEA 10 µM with or without C-C motif chemokine ligand 5 (CCL5) recombinant protein (rh) 20 ng/mL for 24 h, were placed in the top compartments of Boyden chambers (8-μM membranes, Corning Costar, Corning, NY, USA) while a complete medium was added to the bottom within 24 h. Transmigration was quantified as previously described [23].

### 2.5. Invasion Assays

The Matrigel-based invasion assay was performed using chambers coated with Matrigel (Agilent, Bedford, MA, USA 2 mg/mL). TNBC cells were treated with vehicle, DHEA 10 µM, CCL5 rh 20 ng/mL and placed as indicated in Section 2.4. Within 24 h, invaded cells were quantified as reported for transmigration assays.

### 2.6. Metabolic Flux Analysis with the Seahorse XFe96

Real-time oxygen consumption rates (OCRs) and extracellular acidification (ECARs) rates were determined using the Seahorse Extracellular Flux (XFe96) analyzer (Agilent, Santa Clara, CA, USA) [24]. Briefly, 10,000 TNBC cells per well were seeded into XFe96 well cell culture plates, incubated for 24 hours to allow cell attachment, and treated with DHEA 10 µM (and vehicle alone). After 24 h of treatment, cells were washed in pre-warmed XF assay media (or for OCR measurement, XF assay media supplemented with 10 mM glucose, 1 mM Pyruvate, 2 mM L-glutamine, and adjusted at 7.4 pH). Cells were then maintained in 175 μL/well of XF assay media at 37 °C in a non-CO_2_ incubator for 1 hour. During the incubation time, 25 μL of 80 mM glucose, 9 μM oligomycin, and 1 M 2-deoxyglucose (for ECAR measurement) or 10 μM oligomycin, 9 μM FCCP, 10 μM rotenone, 10 μM antimycin A were loaded (for OCR measurement), in XF assay media into the injection ports in the XFe96 sensor cartridge. Measurements were normalized by protein content (SRB assay) [25]. Data sets were analyzed using XFe96 software and GraphPad Prism 7 software, using one-way ANOVA and Student’s *t*-test calculations. All experiments were performed in quintuplicate, three times independently.

### 2.7. Conditioned Medium Systems

MDA-MB-231 and MDA-MB-436 cells were plated in 5% charcoal-stripped serum medium (2.5 × 10^6^) in a 10 cM dish. Then, cells were washed twice and treated with vehicle or DHEA 10 µM for 24 h in a 1% charcoal-stripped serum medium. CM was collected, centrifuged, and used in co-culture experiments.

### 2.8. Macrophage Recruitment

THP-1 cells (10^5^ cells) were added to the upper chamber of a 24-transwell apparatus (5 µm membranes, Corning Costar), while control serum or CM derived from TNBC cells with or without CCL5 rh 20 ng/mL were added to the lower compartment. Migrated cells were fixed and stained with 4′,6-diamidino-2-phenylindole dihydrochloride (DAPI) and quantified by viewing five separate fields per membrane at 10× magnification, using ImageJ (version 1.52t).

### 2.9. RNA Purification and Quantitative Reverse-Transcription Real-Time PCR (qRT-PCR)

Total RNA was extracted using TRIzol (Invitrogen, Breda, The Netherlands), as suggested by the manufacturer, as previously described [26]. cDNA was amplified by qRT-PCR using the master-mix Sensimix SYBR (Bioline Reagents Ltd., London, UK) on a CFX Real-Time System apparatus (Bio-Rad, Veenendaal, The Netherlands). Primers used are listed in Supplementary Table S1. Samples were normalized on 18S rRNA content, and relative gene expression levels were calculated as previously reported [27].

### 2.10. Cytokine Array

Human XL Cytokine Array Kits were used to analyze the secreted proteins in the CM derived from MDA-MB-231 breast cancer cells treated with vehicle or DHEA 10 μM, according to the manufacturer’s recommendations (R&D Systems, Minneapolis, MN, USA). The intensity of selected spots was quantified using Image Studio Lite Version 5.2 (Licor, Lincoln, NE, USA). Results are presented as fold-over to vehicle-treated cells.

### 2.11. Protein–Protein Interaction Network

The analysis of protein-protein interaction was performed by the software Search Tool for the Retrieval of Interacting Genes (STRING) 11.5 [28]. The differentially secreted cytokines were mapped into the STRING database with a combined score > 0.7 for identifying the protein-protein interaction network. The nodes indicate proteins, and the edges indicate the number of interactions. Cytoscape software version 3.9.1 was used to identify the node degree.

### 2.12. Enzyme-Linked Immunosorbent Assay (ELISA)

Levels of CCL5 were measured in supernatants from TNBC cells treated with vehicle or DHEA 10 µM in the presence or not of the irreversible PPARγ antagonist GW9662 (Sigma Aldrich, St. Louis, MO, USA), using human ELISA kits according to manufacturer’s instructions (R&D Systems, Minneapolis, MN, USA).

### 2.13. Immunofluorescence

TNBC cells were treated with vehicle or DHEA 10 µM, with or without MG132 20 µM for 24 h. In another set of experiments, cells were transfected with PPARγ-targeting or control RNA interference, as indicated in Section 2.15 and then treated with DHEA 10 µM for 24 h. Cells were fixed with 4% paraformaldehyde, permeabilized with Phosphate-buffered saline (PBS) + 0.2% Triton X-100 followed by blocking with 5% bovine serum albumin (BSA) for 30 min and incubated overnight with anti-CCL5 antibody (dilution 1:250) in BSA at 4 °C. The day after, the cells were washed three times with PBS and incubated with the secondary antibody anti-mouse IgG-fluorescein isothiocyanate (dilution 1:500) for 1 h at room temperature. To check the specificity of immunolabeling, the primary antibody was replaced by normal mouse serum (negative control). Fluorescence was photographed with an Olympus BX51 microscope (Tokyo, Japan), 100× or 40× objective.

### 2.14. Immunoblot Analysis

Total protein extracts (50 μg) were subjected to SDS-PAGE as described [29]. After blocking, proteins were probed with anti-PPARγ (PA3-821A), anti-phosphatase and tensin homolog PTEN (sc25778) and anti-β-Actin (sc69879) (Santa Cruz Biotechnology, Santa Cruz, CA, USA) antibodies, overnight, and images were acquired using Odyssey FC (Licor, Lincoln, NB, USA).

### 2.15. Stealth RNA Interference (siRNA)

Cells were plated with regular growth medium the day before the transfection to 60–70% confluence, as previously reported [30], and then transfected with a stealth RNA interference (siRNA) targeted human PPARγ mRNA sequence sense: AGAAUAAUAAGGUGGAGAUGCAGGC and antisense: GCCUGCAUCUCCACCUUAUUAUUCU, or with a siRNA negative control (Invitrogen, Carlsbad, CA, USA) to a final concentration of 10 nM using Lipofectamine RNAiMAX (Invitrogen). Reduction in PPARγ expression was measured by both quantitative real-time PCR and immunoblot analysis.

### 2.16. Survival Kaplan-Meier Analysis

Kaplan-Meier analysis was carried out using the Kaplan-Meier plotter online tool by using patient datasets with log-rank tests between groups and the median of expression as a cutoff [31].

### 2.17. Statistical Analysis

All analyses were performed with GraphPad Prism 8 (GraphPad Software, Inc., San Diego, CA, USA). Experimental data were reported as the mean ±SEM. All experiments were conducted at least 3 times independently, with 3 or more technical replicates for each experimental condition tested (unless stated otherwise, e.g., when representative data or figure is shown). Statistically significant differences were determined using the Student’s *t*-test or the analysis of variance (ANOVA) test. For the comparison among multiple groups, one-way ANOVA was used to determine statistical significance. *p* < 0.05 was considered significant.

## 3. Results

### 3.1. The Omega-3 Fatty Acid DHEA Affects Motility and Invasion of TNBC Cells

**Figure 1:**
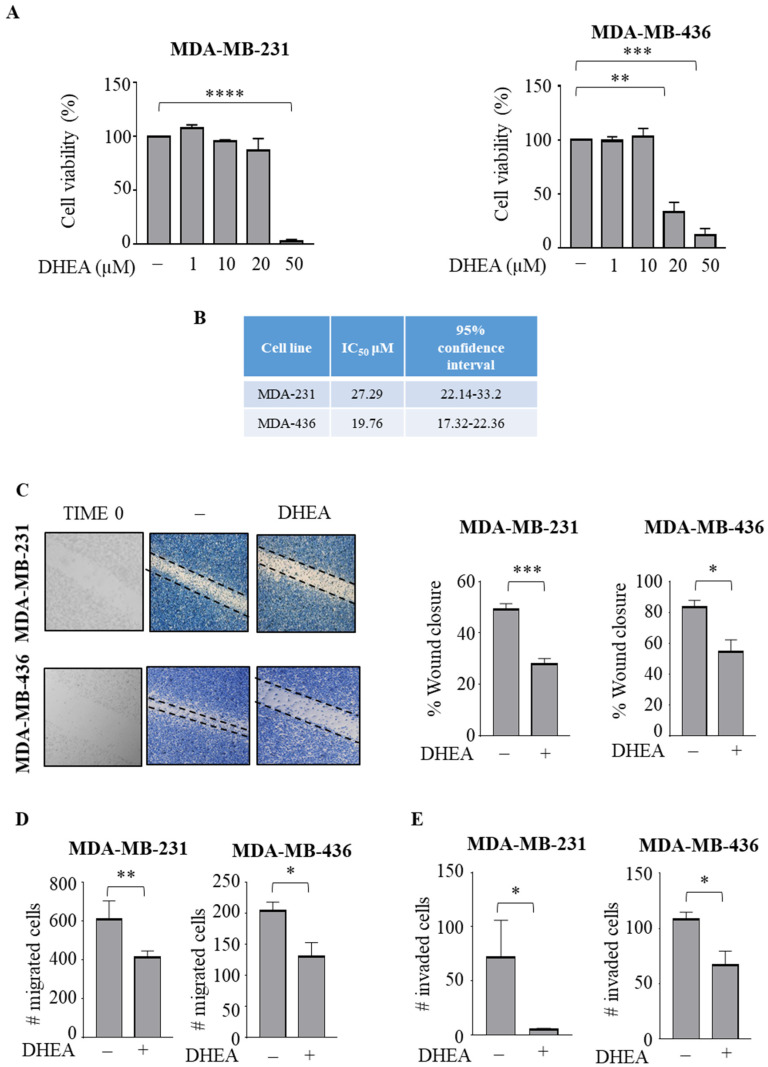
DHEA treatment reduces breast cancer cell motility and invasion. (**A**) MTT (3-(4,5-Dimethylthiazol-2-yl)-2,5-Diphenyltetrazolium Bromide) assay in MDA-MB-231 and MDA-MB-436 breast cancer cells treated as indicated for 24 h. (**B**) IC_50_ values of DHEA in MDA-MB-231 (MDA-231) and MDA-MB-436 (MDA-436) cells from MTT growth assay after 24 h. (**C**) Wound healing assay in MDA-MB-231 and MDA-MB-436 cells treated with vehicle (−) or DHEA 10 µM. Time 0 is shown. Pictures are representative of three independent experiments. Boyden chamber transmigration (**D**,**E**) invasion assays in MDA-MB-231 and MDA-MB-436 cells treated with vehicle (−) or DHEA 10 µM within 24 h. The values represent the mean ±SEM of three different experiments. * *p* < 0.05; ** *p* < 0.005; *** *p* < 0.0005; **** *p* < 0.0001.

First, we examined the effects of the ethanolamide derivative of DHA, docosahexaenoyl ethanolamide (DHEA), on cell viability of both triple-negative MDA-MB-231 and MDA-MB-436 human breast cancer cells. Using MTT assays, we observed that treatment with DHEA for 24 h significantly reduced cell viability at 50 μM in MDA-MB-231 (*p* < 0.0001), with IC50 values of 27.29 μM and exerted its inhibitory effects at lower doses (20 μM) in MDA-MB-436 cells, showing IC_50_ values of 19.76 μM (*p* < 0.005) (Figure 1A,B). We previously demonstrated that DHEA at 10 μM did not elicit any significant growth inhibitory effects in non-tumorigenic MCF-10A breast epithelial cells [32]. Thus, since this compound did not affect cell viability at 10 μM in TNBC cells, for all experiments, this concentration was used to evaluate whether DHEA treatment may cause any changes in cellular phenotype, including migration and invasion, that are crucial characteristics of this aggressive subtype of cancer cells. In wound-healing scratch assays, we found that both TNBC cells treated with DHEA exhibited a reduction in cell movement in either direction to close the gap compared with vehicle (Figure 1C) (*p* < 0.0005 in MDA-MB-231 and *p* < 0.05 in MDA-MB-436). Given the evident decrease in cell motility, the capacity of cells to migrate across an uncoated membrane in transmigration assays or invade an artificial basement membrane in Matrigel invasion assays was tested in the presence of DHEA. Our data clearly showed that DHEA significantly reduced both motility and invasion of MDA-MB-231 (*p* < 0.005 and *p* < 0.05) and MDA-MB-436 (*p* < 0.05 and *p* < 0.05) breast cancer cells (Figure 1D,E).

### 3.2. Metabolic Profiling of TNBC Cell Lines Treated with DHEA

**Figure 2:**
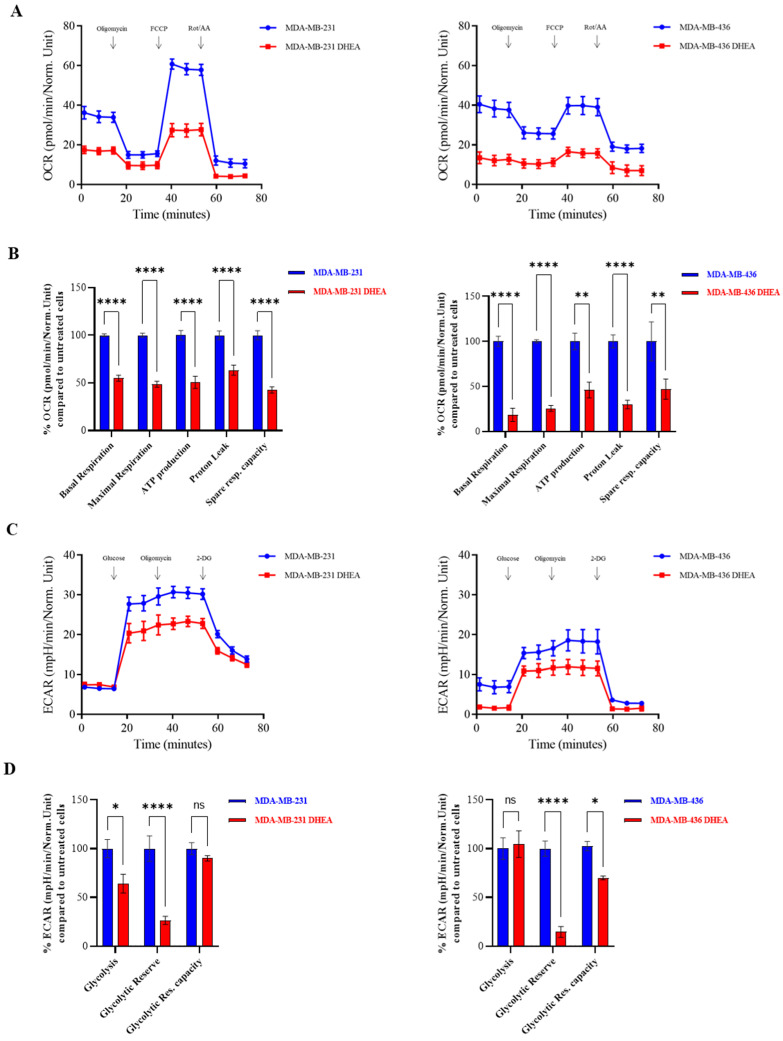
DHEA diminishes oxygen consumption rate and extracellular acidification rate in MDA-MB-231 and MDA-MB-436 breast cancer cells. The Seahorse XF96 analyzer was employed to determine the mitochondrial and glycolytic profile of MDA-MB-231 and MDA-MB-436 breast cancer cells treated with DHEA 10 μM for 24 h. (**A**) OCR profile. (**B**) Respiration (basal and maximal), as well as ATP levels, Proton Leak, and Spare respiratory capacity. (**C**) ECAR profile. (**D**) Glycolysis, Glycolytic Reserve, and Glycolytic Reserve Capacity. The values represent the mean ± SEM of three different experiments. * *p* < 0.01, ** *p* < 0.001, **** *p* < 0.00001, ns = not significant.

Considering that accelerated rates of proliferation, invasion, and migration are sustained by high metabolic rates in malignant breast cancer, we aimed to investigate the impact of DHEA on the metabolic profile of TNBC cells. Real-time oxygen consumption rates (OCR) and extracellular acidification rates (ECAR) for cells treated with DHEA 10 µM were assessed using the Seahorse Extracellular Flux (XFe-96) analyzer. More specifically, OCR is a surrogate marker for OXPHOS activity, while ECAR is a measure of glycolysis. Figure 2A shows that DHEA 10 µM inhibited oxygen consumption in both MDA-MB-231 and MDA-MB-436 cells, reducing respiration, both maximal (*p* < 0.00001) and basal (*p* < 0.00001), as well as ATP levels (*p* < 0.00001 in MDA-MB-231 and *p* < 0.001 in MDA-MB-436), proton leak (*p* < 0.00001) and spare respiratory capacity (*p* < 0.00001 in MDA-MB-231 and *p* < 0.001 in MDA-MB-436) (Figure 2B). In addition, DHEA 10 µM inhibited ECAR in MDA-MB-231 and MDA-MB-436 cells (Figure 2C), reducing the glycolytic reserve in both cell lines (*p* < 0.00001) (Figure 2D). The observed cell energy profile indicated that DHEA is able to shift MDA-MB-231 and MDA-MB-436 cells from an energetic to a quiescent state.

### 3.3. Effects of Conditioned Media from Breast Cancer Cells Treated with DHEA on Macrophage Biology

**Figure 3:**
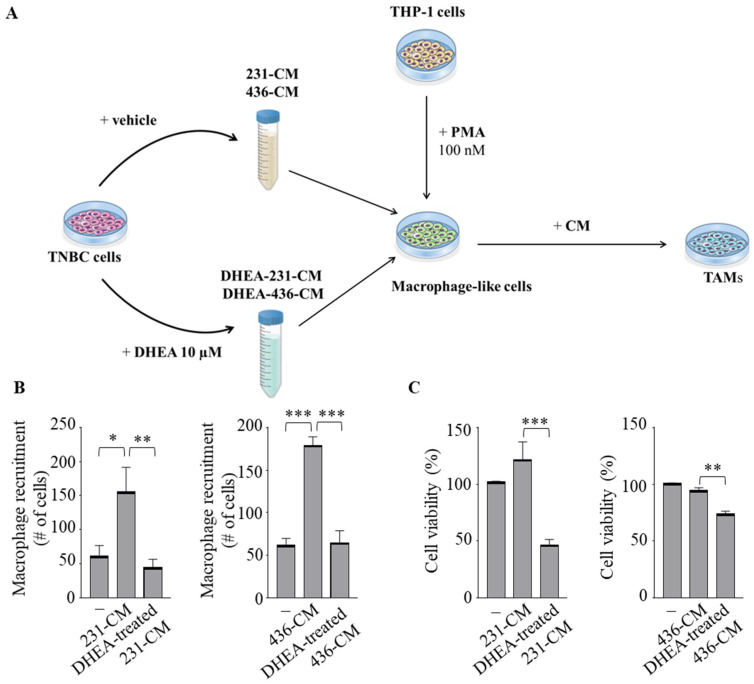

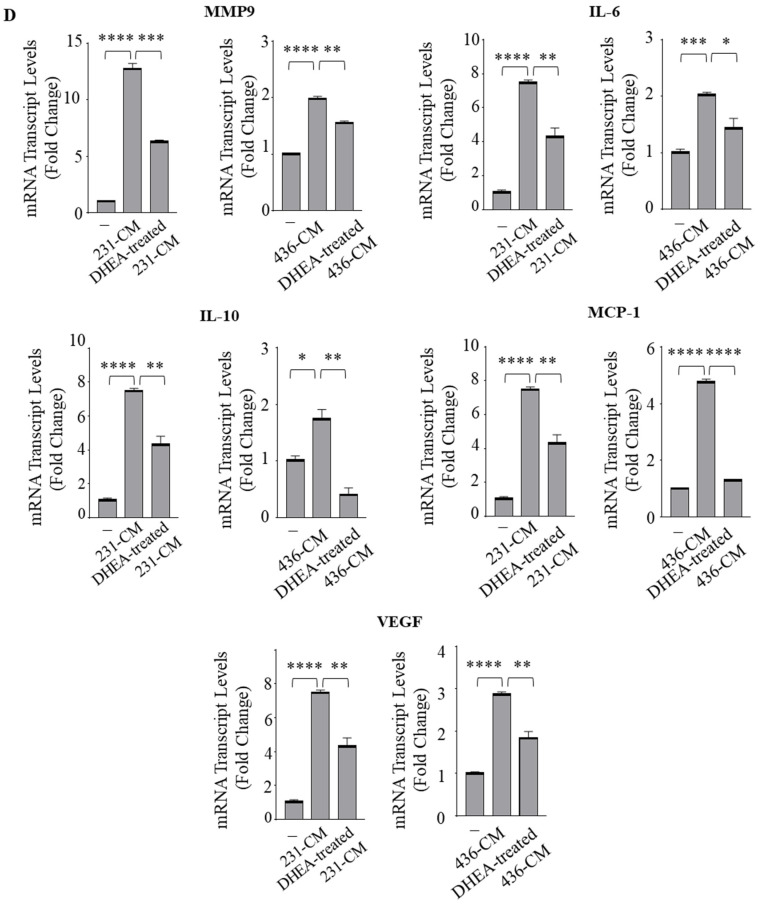
Conditioned media from TNBC cells treated with DHEA modulate macrophage recruitment and Tumor-Associated Macrophage phenotype. (**A**) Schematic representation of the in vitro co-culture experiments to obtain tumor-associated macrophages (TAM). Conditioned media (CM) were collected from both TNBC (MDA-MB-231 and MDA-MB-436) cells treated with vehicle (231-CM and 436-CM) or with DHEA 10 µM for 24 h (DHEA-treated 231-CM and DHEA-treated 436-CM). Human THP-1 monocytes were stimulated with phorbol 12-myristate 13-acetate (PMA, 100 nM) for 14 h followed by 24 h rest to generate macrophage-like cells and then incubated for 5 days with CM as indicated. (**B**) Trans-well recruitment of THP-1 cells in response to control media (−), 231-CM, 436-CM, DHEA-treated 231-CM, or DHEA-treated 436-CM was assessed after 12 h incubation. The migrated monocytes were stained with DAPI, and five random fields were captured per well with an Olympus microscope at 10× magnification. (**C**) MTT (3-(4,5-Dimethylthiazol-2-yl)-2,5-Diphenyltetrazolium Bromide) assay was performed in THP-1-derived macrophages incubated for 5 days with the control media (−), 231-CM, 436-CM, DHEA-treated 231-CM or DHEA-treated 436-CM. (**D**) Real-time qRT-PCR assay for matrix metalloproteinase-9 (MMP-9), interleukin (IL)-6, IL-10, monocyte chemoattractant protein-1 (MCP-1), vascular endothelial growth factor (VEGF) in macrophages incubated as in C. The values represent the mean ± SEM of three different experiments. * *p* < 0.05; ** *p* < 0.005; *** *p* < 0.0005; **** *p* < 0.0001.

Emerging evidence has proven that epithelial-stromal interactions support tumor progression and response to therapy with a poor prognosis for patients [33]. Because macrophages are the principal cellular component of the stroma, we created the in vitro conditions that can mimic the complex breast TME using co-culture experiments with the human monocytic THP-1 cells incubated with conditioned media (CM) collected from both MDA-MB-231 and MDA-MB-436 cell lines treated with vehicle (231-CM or 436-CM) or with DHEA (DHEA-treated 231-CM and DHEA-treated 436-CM), used as chemoattractants (Figure 3A). Interestingly, CM obtained from TNBC cells treated with DHEA reduced the recruitment of monocytes concerning vehicle-treated media (*p* < 0.005 in 231-CM, *p* < 0.0005 in 436-CM) (Figure 3B). Next, to investigate whether DHEA treatment in TNBC cells also modulates the phenotype of TAMs, THP-1 cells were differentiated with PMA into mature macrophage-like cells and incubated for 5 days with CM from TNBC cells untreated or treated with DHEA. Cell viability was significantly decreased when TAMs were cultured with DHEA-treated CM (*p* < 0.0005 in 231-CM, *p* < 0.005 in 436-CM) (Figure 3C). As expected, RT-PCR revealed increased mRNA levels of several genes associated with the TAMs phenotype, such as matrix metalloproteinase-9 (MMP-9), Interleukin (IL)-6, IL-10, MCP-1/C-C motif chemokine ligand 2 (MCP-1/CCL-2), vascular endothelial growth factor (VEGF), in macrophage-like cells cultured with 231-CM and 436-CM with respect to control media (Figure 3D). Remarkably, DHEA-treated CM was able to reverse the mRNA expression induction of these genes compared to CM obtained from both TNBC cell lines (Figure 3D). Collectively, these data indicate that treatment of breast cancer cells with DHEA hampers monocyte recruitment, reduces macrophage cell viability, and attenuates the TAM phenotype.

### 3.4. DHEA Modulates the Release of Chemokine CCL5 from TNBC Cells Affecting Breast Cancer Cell Migration and Macrophage Recruitment

**Figure 4:**
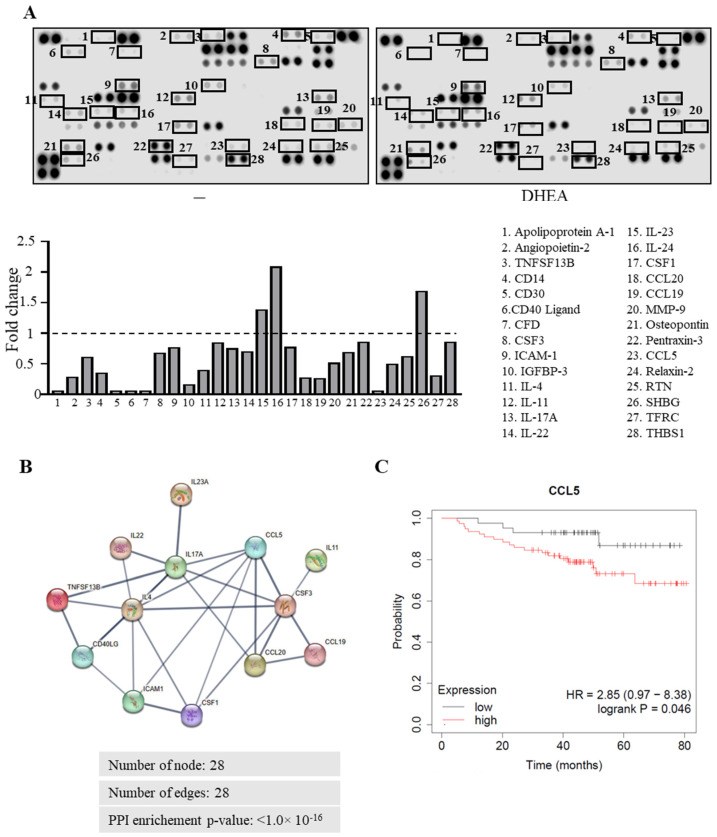
Effects of DHEA on cytokine secretion profile of TNBC cells. (**A**) Human cytokine array of the secreted proteins by MDA-MB-231 breast cancer cells treated with vehicle (−) or DHEA 10 μM. Results are presented as fold-over to vehicle-treated cells. (**B**) Protein–protein interaction (PPI) network visualized by STRING 11.5 online tool (https://string-db.org/, accessed on 28 July 2022). The nodes indicate proteins, and the edges indicate the number of interactions. Disconnected nodes are hidden, and only interactions with a high confidence score of >0.7 are shown. (**C**) Kaplan-Meier survival analysis relating CCL5 levels (using the median of expression as cutoff) and overall survival in TNBC patients. Hazard ratio (HR) and *p*-value are shown.

Cancer cells secrete different soluble factors that may confer plasticity to immune cells, influencing the final outcome of the tumor progression. Thus, to identify the diffusible factor that may be responsible for the effects exerted by DHEA on breast cancer cell secretion, we first compared the cytokine profile of CM from MDA-MB-231 cells treated with DHEA to that treated with vehicle, using a semiquantitative cytokine array and we identified 28 modulated proteins (Figure 4A). Results from analysis of protein–protein interaction evidenced that 13 differentially secreted cytokines were found to be interconnected when analyzed using the STRING web tool in a network with 28 nodes, 29 edges, and a protein-protein interaction *p*-value < 1.0 × 10^−16^ (Figure 4B). Interleukin (IL)-17A, IL-4, Colony Stimulating Factor (CSF)3, and C-C motif chemokine ligand 5 (CCL5) were found to be the cytokines showing the highest degree using Cytoscape software (Supplementary Table S2). Moreover, analyzing the expression of these proteins in our array, we observed that CCL5 was the most downregulated cytokine by DHEA. Previous literature data reported that CCL5, but not the other three cytokines, is highly secreted by breast cancer cells compared to normal cells [34]. Interestingly, the Kaplan Meier plotter (http://kmplot.com/, accessed on 10 June 2022) [35] showed that a low CCL5 expression predicted a higher overall survival in TNBC patients (HR = 2.85: 0.97–8.38; *p* = 0.046) (Figure 4C). Thus, we focused our attention on the potential involvement of CCL5 in the effects exerted by DHEA in TNBC cells.

**Figure 5:**
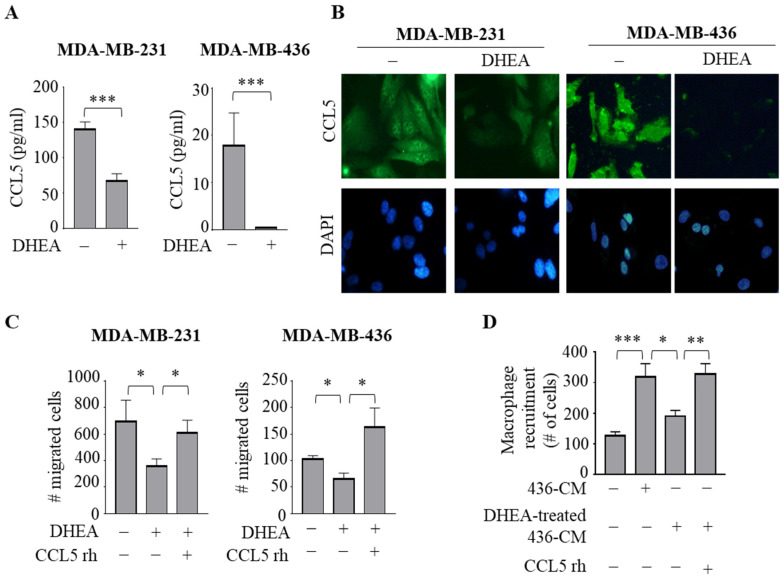
DHEA reduces the expression and secretion of the CCL5 in TNBC cells. (**A**) ELISA for C-C Motif Chemokine Ligand (CCL5) protein secretion in MDA-MB-231 and MDA-MB-436 breast cancer cells treated with vehicle (−) or DHEA 10 μM for 24 h. (**B**) Immunofluorescent staining of CCL5 protein expression in MDA-MB-231 and MDA-MB-436 treated as in (**B**) DAPI staining was used for nuclei detection (inset). (**C**) Boyden chamber transmigration assays in cells treated with vehicle (−), DHEA 10 µM with or without CCL5 recombinant protein (rh) 20 ng/mL for 24 h. (**D**) Trans-well recruitment of THP-1 cells in response to 1% charcoal-stripped media (−) and the conditioned medium (CM) derived from MDA-MB-436 breast cancer cells treated with vehicle (436-CM) or with DHEA 10 µM (DHEA-treated 436-CM) with or without CCL5 rh 20 ng/mL. * *p* < 0.05; ** *p* < 0.005; *** *p* < 0.0005.

To validate the results of the cytokine array analysis, we analyzed the secretion of CCL5 in the collected CM from both TNBC cells treated with DHEA by ELISA. Interestingly, protein levels of CCL5 were 143.47 ± 15.05 pg/mL and 17.9 ± 6.88 pg/mL in MDA-MB-231 and MDA-MB-436 cell-derived CM, respectively. Compared to basal levels, DHEA treatment markedly reduced CCL5 concentrations at 51.3 ± 2.84 pg/mL and 0.5 ± 0.01 pg/mL in MDA-MB-231 and MDA-MB-436 cell-derived CM, respectively (*p* < 0.0005) (Figure 5A). Accordingly, the reduction in CCL5 expression induced by DHEA in both TNBC cells was confirmed by evaluating protein levels using immunofluorescent staining (Figure 5B).

Having previously demonstrated that ethanolamide derivative DHEA modulates PPARγ expression and activity in MCF-7 breast cancer cells [21], we investigated the involvement of this receptor in the effects of DHEA on TNBC cells. As expected, DHEA acts as a PPARγ activator since we found in cells treated with DHEA an enhanced expression of PPARγ at mRNA levels using RT-PCR and an increased expression of protein phosphatase and tensin homolog on chromosome ten (PTEN), which is a target gene of PPARγ by using immunoblotting analysis (Supplementary Figure S1A,B). To establish if PPARγ activated by DHEA is responsible for CCL5 downregulated secretion in our cell system, cells were treated with the irreversible PPARγ antagonist GW9662, which did not reverse the inhibitory effect exerted by DHEA, addressing that this molecule acts in a PPARγ-independent manner (Supplementary Figure S1C). Moreover, to further ascertain the role played by this receptor, RNA silencing technologies were used to knock down the expression of endogenous PPARγ in MDA-MB-231 cells. PPARγ expression was effectively silenced, as revealed by RT-PCR and immunoblotting analyses (Supplementary Figure S1D). However, silencing of PPARγ did not abrogate the down-regulation of CCL5 induced by DHEA (Supplementary Figure S1E), confirming that the effects on CCL5 expression were independent of PPARγ activation.

Since the effects of recombinant human CCL5 (CCL5 rh) on breast cancer progression have been reported [36], as the final step of this study, we aimed to prove whether the action of DHEA occurs through CCL5. Thus, we analyzed the effects of DHEA on breast cancer cell migration as well as of DHEA-treated CM from TNBC cell lines on macrophage recruitment in the presence of CCL5 rh. We observed that this chemokine reversed the inhibitory properties of DHEA in breast cancer cells and in macrophages (Figure 5C,D), suggesting that disruption of this breast cancer cell-secreted factor may modulate both neoplastic epithelial cells and the reactive immune surrounding cell population leading to a tumor-suppressing microenvironment.

## 4. Discussion

In this study, we showed that the conjugate of the omega-3 DHA with ethanolamine, DHEA, affects the aggressiveness of TNBC cells impairing their interaction with the surrounding immune tumor microenvironment. These data open new insight into the antitumoral action of DHEA and encourage an investigation of the efficacy of this compound in the management of TNBC.

The treatment of TNBC is complex and challenging due to its aggressive behaviors, which determine a high rate of therapeutic treatment failure. Thus, there is an urgent need to find new strategies for the treatment of this type of breast cancer. Natural compounds have been studied for their potential benefits in cancer prevention and treatment. In particular, the omega-3 PUFAs, DHA and EPA, have been found to lower risk and counteract the development of certain types of malignancy, including breast cancer. Particularly, it has been demonstrated that DHA and EPA induced apoptosis in breast cancer cells, diminished tumor angiogenesis, and increased the efficacy of chemotherapeutic agents. Moreover, clinical studies have revealed that DHA and EPA administration reduced the side effects of chemotherapy [14,37,38,39]. Thus, omega-3 PUFA supplementation could be of value for patients with TNBC. However, the mechanisms of action of these compounds in breast cancer are still poorly understood and must be extensively investigated. It has been demonstrated that the effects of omega-3 PUFAs depend, at least in part, on the conversion into their metabolites, including ethanolamine derivatives. DHEA, a more active derivative of the omega-3 DHA, has been found in the plasma of healthy volunteers upon daily supplementation with fish oil food, suggesting that the amount of ethanolamide conjugates is related to the dietary intake of omega-3 PUFAs [40]. Moreover, the formation of N-acylethanolamines upon administration of omega-3 PUFAs has also been found in human adipocytes, hippocampal neuron cultures, and different cancer cells, including human breast cancer cells, highlighting the need to unravel the potential anti-tumoral action of this compound for breast cancer treatment [19,41,42]. In our previous work, we found that DHEA at the concentration of 10 μM exerted anti-proliferative effects in estrogen receptor-positive MCF-7 breast cancer cells without affecting the cell viability of normal mammary epithelial MCF-10A cells [32]. These results are in accordance with the data of Brown’s study, showing that higher concentrations of DHEA did not affect the viability of MCF-10A cells [43]. In the present study, investigating the action of DHEA in TNBC cells, we did not observe reduced cell viability in both TNBC cells upon treatment with DHEA at 10 μM, probably due to their increased proliferative behaviors. In contrast, higher concentrations of DHEA significantly elicited antiproliferative responses with IC_50_ values of 27.29 μM and 19.76 μM in MDA-MB-231 and MDA-MB-436 breast cancer cells, respectively. Based on these data, we studied whether non-toxic concentrations of DHEA may affect the phenotypic properties of TNBC cells. One of the most important characteristics of TNBC cells is their high motility, which gives them the ability to move through and invade surrounding tissues and organs prior to diagnosis, establishing metastasis in bones, brain, lungs, liver, and lymph nodes [44]. Although metastatic TNBC is initially responsive to chemotherapy, which represents the first-line treatment for metastatic TNBC, it has been widely demonstrated that it quickly becomes resistant to pharmaceutic treatment leading to patient death [45]. In our study, we found that DHEA reduced MDA-MB-231 and MDA-MB-436 breast cancer cell migration and invasion, suggesting that this compound may counteract the development of breast cancer metastasis. In contrast, Brown et al. have found that DHEA reduced only the invasiveness of TNCB cells without affecting breast cancer cell migration [43]. In addition, we found that DHEA affected the metabolic profile of TNBC cells, causing a dramatic inhibition of both oxygen consumption and ATP production, conferring reduced survival advantages on breast cancer cells. Moreover, DHEA treatment reduced the glycolytic reserve levels, switching both cell lines from an energetic to a quiescent state.

Breast cancer development, progression, and treatment resistance are also influenced by the interaction between tumor cells and their microenvironment. It has been well established that during tumor initiation, breast cancer cells educate the surrounding non-malignant cells in the TME to acquire a new phenotype that promotes tumorigenesis. In turn, the transformed cells in TME release cytokines, chemokines, growth factors, inflammatory mediators, and matrix remodeling enzymes, creating a favorable milieu for tumor progression [46,47,48,49]. Among stromal cells, TAMs representing over 50% of the tumor mass have been shown to correlate with tumor aggressiveness and predict poorer prognosis in almost all tumors, including breast carcinoma [6]. Studies on macrophage and cancer cell interactions have emphasized that TAMs are plastic cells with different functions and cytokine production in response to various micro-environmental switching signals [50,51]. Thus, strategies aiming to target TAMs promise therapeutic benefits. In particular, it has been proposed to inhibit TAM infiltration or reprogram their phenotype in order to activate their anti-tumor functions [52]. In our study, we found that the conditioned media of TNBC cells treated with DHEA reduced monocyte recruitment and TAM viability with respect to control, suggesting the potential use of DHEA to block macrophage recruitment and increase TAM depletion in the breast TME. Of note, since macrophages mediate the host defense, homeostasis, and erythropoiesis, further investigations are needed to evaluate whether DHEA selectively induces a specific TAM depletion. Interestingly, we demonstrated that DHEA educated TAMs in a less aggressive phenotype, reducing the gene expression of several markers of TAM phenotype, including IL-6, MMP-9, VEGF, IL-10, and MPC-1, which sustain tumor invasiveness, angiogenesis, and metastasis [53]. Altogether, these data highlight the potential role of DHEA in acting on both stromal and tumor cells, affecting breast cancer progression.

Within the TME, paracrine communication between cells mainly occurs through soluble factors such as hormones, cytokines, and growth factors. Our data showed that DHEA modulated the expression of 28 proteins secreted by TNBC cells. Among these, only 13 cytokines created a protein-protein interaction network in which CCL5, IL-4, IL-17A, and CSF-3 represent key factors. Based on data reported in the literature [34], CCL5 expression is increased in TNBC cell lines compared to non-tumorigenic breast cancer cells, whereas similar expression of IL-4, IL-17A, and CSF-3 have been found between malignant and normal breast epithelial cells, indicating the crucial role of CCL5 in breast tumorigenesis. We found that DHEA completely abrogated the secretion of CCL5 by breast cancer cells, suggesting that this cytokine may mediate the anti-tumoral properties of DHEA in TNBC cell lines. The mechanism by which DHEA reduced CCL5 secretion is not fully elucidated. Although it has been reported that DHEA is an agonist of PPARγ [21], our data demonstrated that its effect is independent of receptor activation.

The role of CCL5 in sustaining cancer progression is well-established [54]. CCL5 is an inflammatory chemokine that modulates the phenotype of both epithelial and stromal cells. In particular, increased expression of CCL5 has been found to be associated with a more aggressive and invasive breast cancer phenotype [55]. Recently, it has been observed that the expression of CCL5 is strongly associated with breast cancer progression, especially in TNBC [56]. In the TME, it has been reported that CCL5 promotes the migration of endothelial cells, supports angiogenesis [57], and increases the recruitment of CCR5+ monocytes and their polarization into TAM [58,59]. Interestingly, we found that the addition of CCL5 recombinant protein in the conditioned media of breast cancer cells treated with DHEA rescued monocyte recruitment and, in an autocrine manner, TNBC cell migration.

Overall, although future investigations using animal models will be useful to strengthen our results, the present study highlights a novel tumor suppressive role played by DHEA through CCL5 reduction in malignant breast cancer. Since higher expression of CCL5 is associated with poorer overall survival in patients with breast cancer, this chemokine may be an attractive target of DHEA in adjuvant therapy for improving clinical care and decreasing mortality from TNBC patients.

## 5. Conclusions

Our results highlight the effects of DHEA as a potential anti-cancer agent in TNBC by targeting the chemokine CCL5. Although further research is required to validate DHEA for clinical use, our findings indicate this molecule as a potential adjuvant for TNBC treatment, able to affect both epithelial breast cancer cells and macrophages in their microenvironment, reducing tumor aggressiveness.

## Data Availability

The data presented in this study are available on request from the corresponding author.

## Supplementary Materials

The following supporting information can be downloaded at: https://www.mdpi.com/article/10.3390/cancers15030819/s1, Figure S1: The effects of DHEA on the C-C Motif Chemokine Ligand 5 expression are Peroxisome Proliferator-Activated Receptor gamma independent in MDA-MB-231 breast cancer cells; Table S1: Oligonucleotide primers used in the study; Table S2: List of cytokines and node degree identified by Cytoscape (software version 3.9.1).
