# Supplementary material for: The Omega-3 Docosahexaenoyl Ethanolamide Reduces CCL5 Secretion in Triple Negative Breast Cancer Cells Affecting Tumor Progression and Macrophage Recruitment
Source: Cancers (Basel). 2023 Jan 29;15(3):819. doi: 10.3390/cancers15030819 (PMC9913844; doi:10.3390/cancers15030819)
Supplement: Supplementary file 1 [file cancers-15-00819-s001.zip › cancers-2164524-supplementary/Supplementary Figure.pdf]

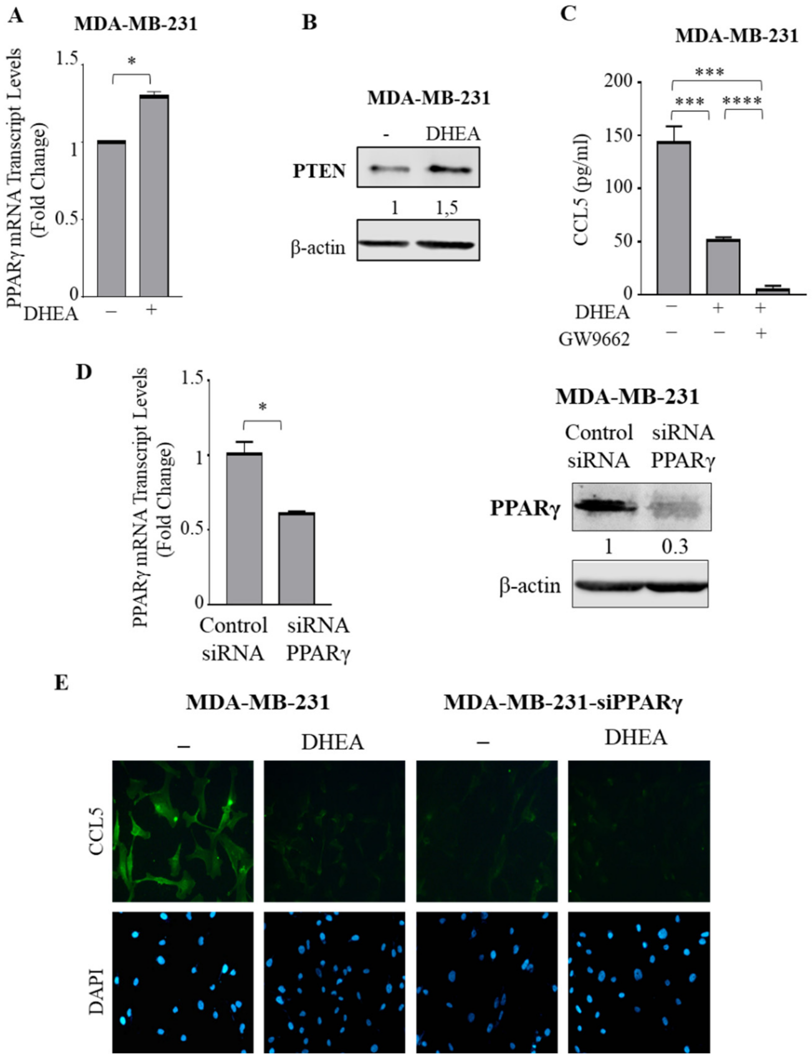

Supplementary Figure S1. The effects of DHEA on the C-C Motif Chemokine Ligand 5 expression and secretion are Peroxisome Proliferator-Activated Receptor gamma-independent in MDA-MB-231 breast cancer cells. Real-time RT-PCR assay (**A**) and immunoblotting analysis (**B**) for Peroxisome Proliferator-Activated Receptor gamma (PPARγ) in MDA-MB-231 breast cancer cells treated with vehicle (−) or DHEA 10 mM for 24 hours. (**C**) ELISA for CCL5 protein secretion in MDA-MB-231 breast cancer cells treated with vehicle (−) or DHEA 10 μM for 24 hours in presence or not of the PPARγ antagonist GW9662. (**D**) Real-time RT-PCR assay and immunoblotting analysis for PPARγ in MDA-MB-231 cells transfected with control stealth RNA (Control siRNA) or PPARγ RNAi (siRNA PPARγ). (**E**) Immunofluorescent staining of CCL5 protein expression in MDA-MB-231 breast cancer cells transfected as in D and treated with

vehicle (−) or DHEA 10 μM for 24 hours. 4',6-diamidino-2-phenylindole dihydrochloride (DAPI) staining was used for nuclei detection. *p<0.05; ***p<0.005, ****p<0.0001.
