# Supplementary material for: The Omega-3 Docosahexaenoyl Ethanolamide Reduces CCL5 Secretion in Triple Negative Breast Cancer Cells Affecting Tumor Progression and Macrophage Recruitment
Source: Cancers (Basel). 2023 Jan 29;15(3):819. doi: 10.3390/cancers15030819 (PMC9913844; doi:10.3390/cancers15030819)
Supplement: Supplementary file 1 [file cancers-15-00819-s001.zip › cancers-2164524-supplementary/Supplementary Table S1.pdf]

Table S1. Oligonucleotide primers used in the study.

Gene Name	Gene Symbol		Primer Sequences
18s rRNA	18s rRNA	Forward	5′-CCCACTCCTCCACCTTTGAC-3′
		Reverse	5′-TGTTGCTGTAGCCAAATTCGTT-3′
Monocyte Chemoattractant Protein-1	MCP-1	Forward	5′-CAGCCAGATGCAATCAATGCC-3′
		Reverse	5′-TGGAATCCTGAACCCACTTCT-3′
Metalloproteinase 9	MMP-9	Forward	5′-AGTTCCCGGAGTGAGTTGAA-3′
		Reverse	5′-CTCCACCCTCCCTTTCCTC-3′
Vascular Endothelial Growth Factor	VEGF	Forward	5'-GAGATGAGCT TCCTACAGCAC-3′
		Reverse	5'-TCACCGCCTCGGCTTGTCACAT-3′
Interleukin 6	IL-6	Forward	5′-CCAGGAGCCCAGCTATGAAC-3′
		Reverse	5′-CCCAGGGAGAAGGCAACTG-3′
Interleukin 10	IL-10	Forward	5′-TCTCCGAGATGCCTTCAGCAGA-3′
		Reverse	5′-TCAGACAAGGCTTGGCAACCCA-3′
Peroxisome Proliferator-Activated Receptor gamma	PPAR	Forward	5′-AACTCAAACTTGGGCTCCATAA AG -3′
		Reverse	5′-CCCACTCCTCCACCTTTGAC-3′
