# Supplementary material for: The Omega-3 Docosahexaenoyl Ethanolamide Reduces CCL5 Secretion in Triple Negative Breast Cancer Cells Affecting Tumor Progression and Macrophage Recruitment
Source: Cancers (Basel). 2023 Jan 29;15(3):819. doi: 10.3390/cancers15030819 (PMC9913844; doi:10.3390/cancers15030819)
Supplement: Supplementary file 1 [file cancers-15-00819-s001.zip › cancers-2164524-supplementary/Supplementary Table S2.pdf]

Table S2. List of cytokines and node degree identified by Cytoscape (software version 3.9.1).

NAME	DESCRIPTION	DEGREE
IL17A	Interleukin-17A	8
IL4	Interleukin-4	8
CSF3	Colony Stimulating Factor 3	7
CCL5	Chemokine (C-C motif) Ligand 5	6
CCL20	Chemokine (C-C motif) Ligand 20	4
CD40LG	Cluster of Differentiation 40 ligand	4
CSF1	Colony Stimulating Factor 1	4
ICAM1	Intercellular Adhesion Molecule 1	4
TNFSF13B	TNF Superfamily Member 13b	3
CCL19	Chemokine (C-C motif) Ligand 19	2
IL22	Interleukin-22	2
CFD	Complement Factor D	1
IL11	Interleukin-11	1
IL23A	Interleukin-23A	1
RETN	Resistin	1
SPP1	Secreted Phosphoprotein 1	1
THBS1	Thrombospondin-1	1
ANGPT2	Angiopoietin-2	0
APOA1	Apolipoprotein A1	0
CD14	Cluster of Differentiation 14	0
IGFBP3	Insulin-Like Growth Factor Binding Protein-3	0
IL24	Interleukin-24	0
PTX3	Pentraxin 3	0
RLN2	Relaxin-2	0
SHBG	Sex Hormone-Binding Globulin	0
TFRC	Transferrin Receptor	0
TIMP4	Tissue Inhibitor of Metalloproteinase-4	0
TNFRSF8	TNF Receptor Superfamily Member 8	0
